# DAYS AT DUNROVIN: ANALYSIS OF FIELD NOTES FROM AN ONLINE NATURE-BASED INTERVENTION FOR OLDER ADULTS

**DOI:** 10.1093/geroni/igad104.3257

**Published:** 2023-12-21

**Authors:** Megan Westmore, Anna Tulloh, Rebecca Mauldin, Keith Anderson, Aaron Hagedorn

**Affiliations:** The University of Texas at Arlington, Arlington, Texas, United States; The University of Texas at Arlington, Arlington, Texas, United States; The University of Texas at Arlington, Arlington, Texas, United States; University of Mississippi, Oxford, Mississippi, United States; The University of Texas at Arlington, Arlington, Texas, United States

## Abstract

Implementing novel interventions for older adults is often challenging, and understanding processes is as important as evaluating outcomes. Days at Dunrovin (D@D) is an online program providing virtual access to a Montana ranch. While originally designed as an individual activity, researchers converted D@D into a group activity for older adults. In this presentation, researchers present an analysis of field notes from 65 group sessions (n = 65) in an assisted living community. Results are organized using the first four elements of the RE-AIM framework (Reach, Effectiveness, Adoption, Implementation, and Maintenance). Reach (i.e., participation) depended on encouraging participation and transporting residents to activity areas – often time-consuming, and diminished by limited resources. Effectiveness (preliminary) was evidenced by participant engagement with the activity, and by staff and family observations of participants’ affect and behavior. Laugher, conversations, and reminiscence were consistently observed. Adoption, the level of facility acceptance, was mixed as some staff welcomed the new activity while others found it difficult to support while attending to regular work. Implementation depended on many factors, including the environment (e.g., room arrangement), time of day, fluctuations in participant well-being, and technological capacities such as broadband width. Most notable was a lightning strike on the D@D cameras that resulted in the temporary use of archival content rather than viewing livestreams. Results suggest the feasibility and potential effectiveness of using nature-based livestream broadcasts as a group activity in assisted living and reveal the invaluable role field notes play in understanding implementation and evaluating interventions in applied research.

